# Suprapatellar pouch effusion is associated with an increased risk of neglected osteochondral fractures in primary acute traumatic patellar dislocation: a consecutive series of 113 children

**DOI:** 10.1186/s13018-023-04130-8

**Published:** 2023-08-26

**Authors:** Mingyuan Miao, Haoqi Cai, Zhigang Wang, Liwei Hu, Jingxia Bian, Haiqing Cai

**Affiliations:** 1 Department of Orthopedic Surgery, Shanghai Children’s Medical Center, School of Medicine, Shanghai Jiao Tong University, Shanghai, China; 2 Department of Radiology, Shanghai Children’s Medical Center, School of Medicine, Shanghai Jiao Tong University, Shanghai, China

**Keywords:** Primary acute traumatic patellar dislocation, Neglected osteochondral fractures, Children, Suprapatellar pouch effusion

## Abstract

**Background:**

The aim of this study was to investigate the risk factors of neglected osteochondral fractures in primary acute traumatic patellar dislocation in the pediatric population.

**Methods:**

A total of 113 patients with primary acute traumatic patellar dislocation for whom coincident osteochondral fractures could not be confirmed by X-ray examination at initial diagnosis between January 2010 and February 2022 were retrospectively analyzed. Medical history, physical examination, and radiographic images were recorded in detail. The greatest dimension of the suprapatellar pouch (SP) effusion on radiograph was measured. Computed tomography and magnetic resonance imaging were used to confirm the presence of neglected osteochondral fractures and measure the fragment size. Potential risk factors were calculated and correlated with reference to the neglected osteochondral fractures and fragment size using multivariate linear regression analysis.

**Results:**

Weight, walking ability, effusion grade, and SP measurement had a significant correlation with neglected osteochondral fractures in primary acute traumatic patellar dislocation (*p* = 0.046; *p* < 0.001; *p* = 0.048; *p* < 0.001). The cutoff point was 53.5 kg for weight and 18.45 mm for SP measurement. In the neglected fractures group, SP measurement was statistically significant with larger fragment size (beta value = 0.457; *p* < 0.001), and the cutoff point was 26.2 mm.

**Conclusions:**

SP effusion is not only associated with an increased risk of neglected osteochondral fractures in primary acute traumatic patellar dislocation but also with larger fragment size. Knee radiograph, medical history, and physical examination can predict the need for further imaging examination and even surgery in primary acute traumatic patellar dislocation.

## Introduction

Acute traumatic patellar dislocation (ATPD) is one of the most common knee injuries. ATPD accounts for 2–3% of acute knee injuries and adolescents have the highest incidence [1, 2]. The recurrence rate of the first dislocation after conservative treatment is about 27% to 43% [3, 4]. Some patients develop long-term complications in adulthood, such as pain, instability, focal chondral disease, and osteoarthritis [5, 6]. Therefore, when ATPD first occurs, accurate diagnosis and treatment are very important. Primary (first-time) ATPD is defined as a traumatic patella disruption of the previously uninjured medial peripatellar structures. However, there is no clear consensus on clinical management. A conservative approach in the absence of an osteochondral fragment seems to be the first choice, but surgery seems necessary when obvious osteochondral fractures (OCFs) are present [7, 8]. The existence of OCFs and fragment size often play a vital role in treatment decision-making. OCFs often indicate more serious injury and the need for more active treatment.

**Fig. 1:**
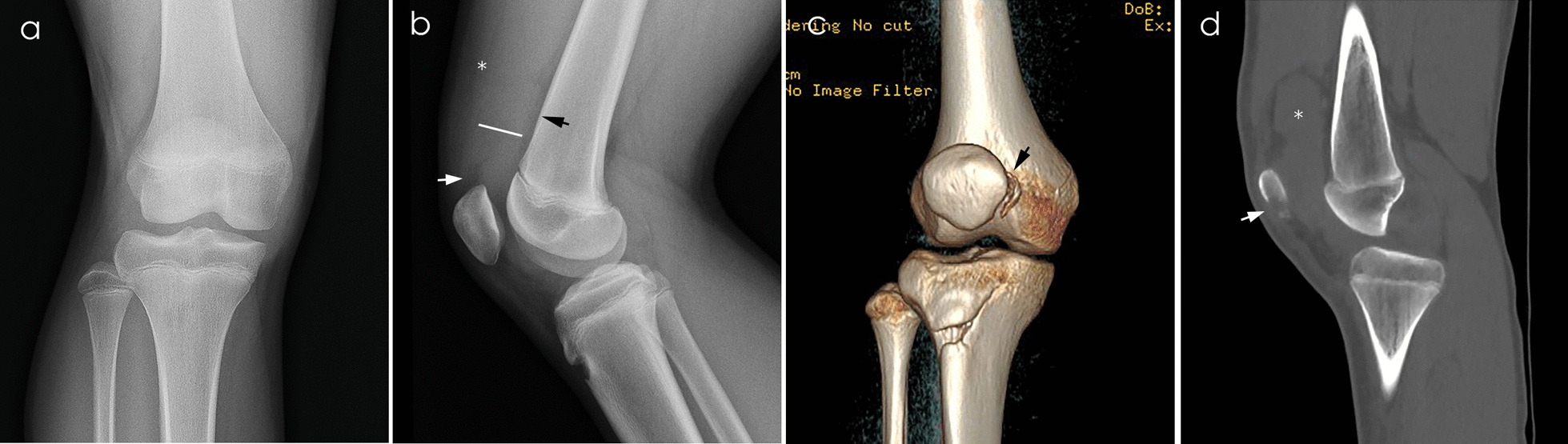
Plain radiograph and CT of a 13-year-old boy with right traumatic patellar dislocation. **a** Anteroposterior knee radiograph. **b** Lateral knee radiograph. The quadriceps fat (white arrowhead) and prefemoral fat (black arrowhead) are deep to the quadriceps tendon (star). SP effusion was measured with a fluid stripe (white line) intervening between the prefemoral fat and quadriceps fat. **c** CT after reconstruction showing fracture fragment at the medial inferior edge of the patella (black arrowhead). **d** Sagittal view of CT showing fracture line (white line) and obvious SP pouch effusion (star)

**Fig. 2:**
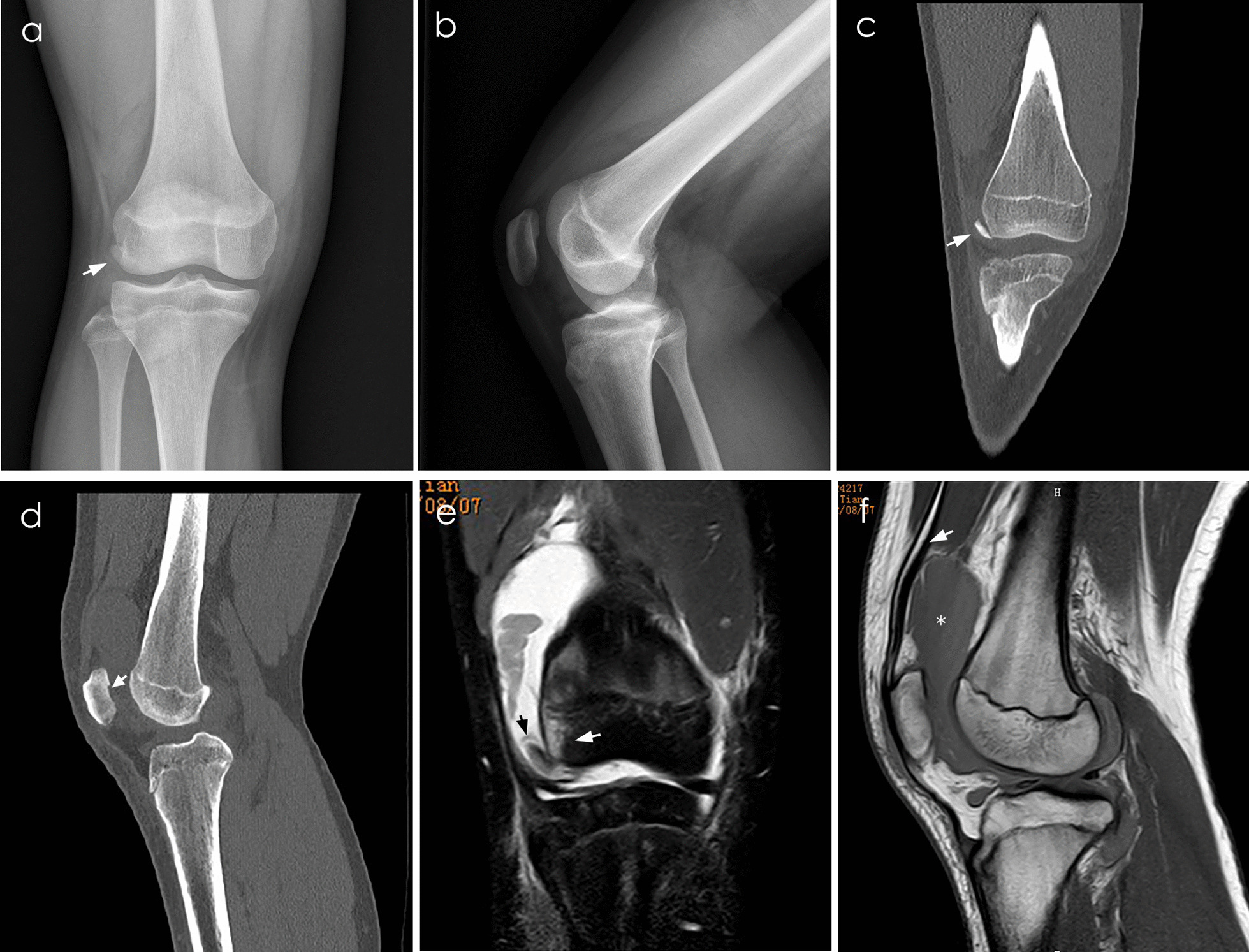
Plain radiograph, CT, and MRI of a 14-year-old boy with right traumatic patellar dislocation. **a** Anteroposterior knee radiograph shows the fracture fragment overlapping with the femoral lateral condyle, which resulted in a missed diagnosis. **b** Lateral knee radiograph. **c** Coronal view of CT showing the fracture fragment clinging to the femoral lateral condyle (white arrowhead). **d** Sagittal view of CT showing patella articular defect lesion (white arrowhead). **e** T2-weighted MRI showing fracture fragments in the lateral sulcus of the knee (black arrowhead) and contusion of the lateral condyle of the femur (white arrowhead). **f** T1-weighted MRI showing obvious SP pouch effusion (star) and quadriceps tendon (white arrowhead)

**Fig. 3:**
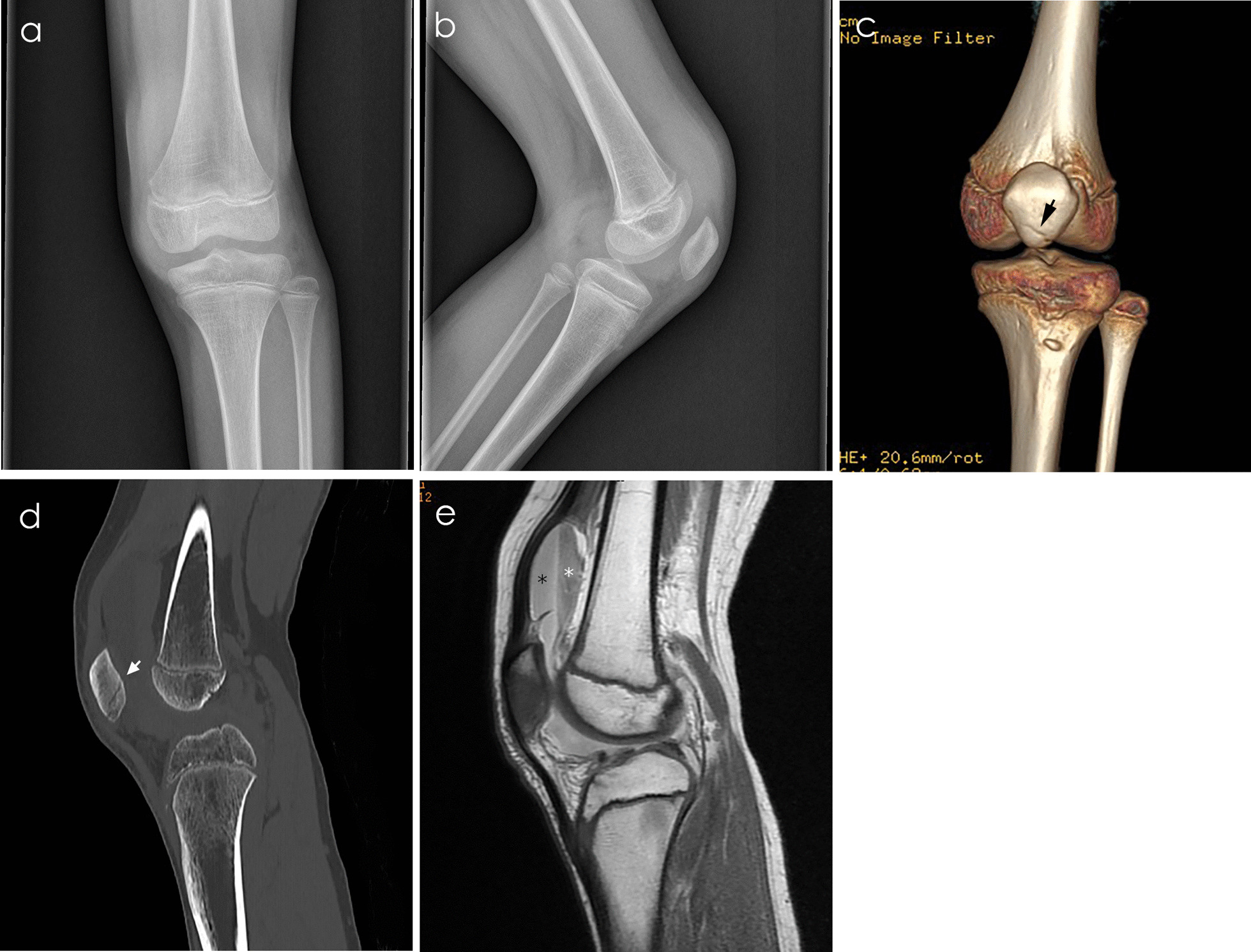
Plain radiograph, CT, and MRI of a 12-year-old boy with right traumatic patellar dislocation. **a, b** Anteroposterior and lateral knee radiograph did not show the patella fracture line. **c** CT after reconstruction showed the fracture line at the inferior pole of the patella (black arrowhead). **d** Sagittal view of CT showing patellar line running through the anterior and posterior edges of the patella (white arrowhead). **e** SP pouch lipohemarthrosis in T1-weighted MRI. Images show a superior band of fat (black star) and dependent band of red blood cells (white star)

Previous studies have reported an OCF incidence of 39% to 71% after ATPD [9]. However, knee radiographs provide limited information because it is frequently difficult to see knee OCFs due to the shelter from the lower femur/patella and their small size (Figs. 1, 2, 3) [10–12]. OCFs and fragment size often need to be confirmed by computed tomography (CT) and magnetic resonance imaging (MRI). However, CT-related radiation exposure increases tumor risk in children [13]. Another consideration is that ionizing radiation in growing children is more likely to occur in proliferating cells [14]. MRI has the benefit of avoiding radiation exposure; however, it also has the obvious disadvantages of higher costs and lower availability [15]. Some scholars advise that attention should be paid to pediatric CT and MRI overuse trends [16]. Therefore, it is important to determine the risk factors of the existence of OCFs in primary ATPD.

One of the common clinical findings is knee hemarthrosis, caused by rupture of the medial restraints of the patella, and chondral injury [17, 18]. As the injury occurs on the patellofemoral joint, the knee swelling is often the most obvious at the SP. Physical examination of knee swelling and imaging measurement of SP have been used to evaluate knee injury [19]. Lateral knee radiographs are highly sensitive for the detection of knee effusion and offer high accuracy and specificity when SP measurement is more than 7 mm [20]. SP measurement greater than 10 mm should prompt consideration for magnetic resonance examination, which may decrease delayed diagnosis and improve patient outcomes [21].

**Fig. 4:**
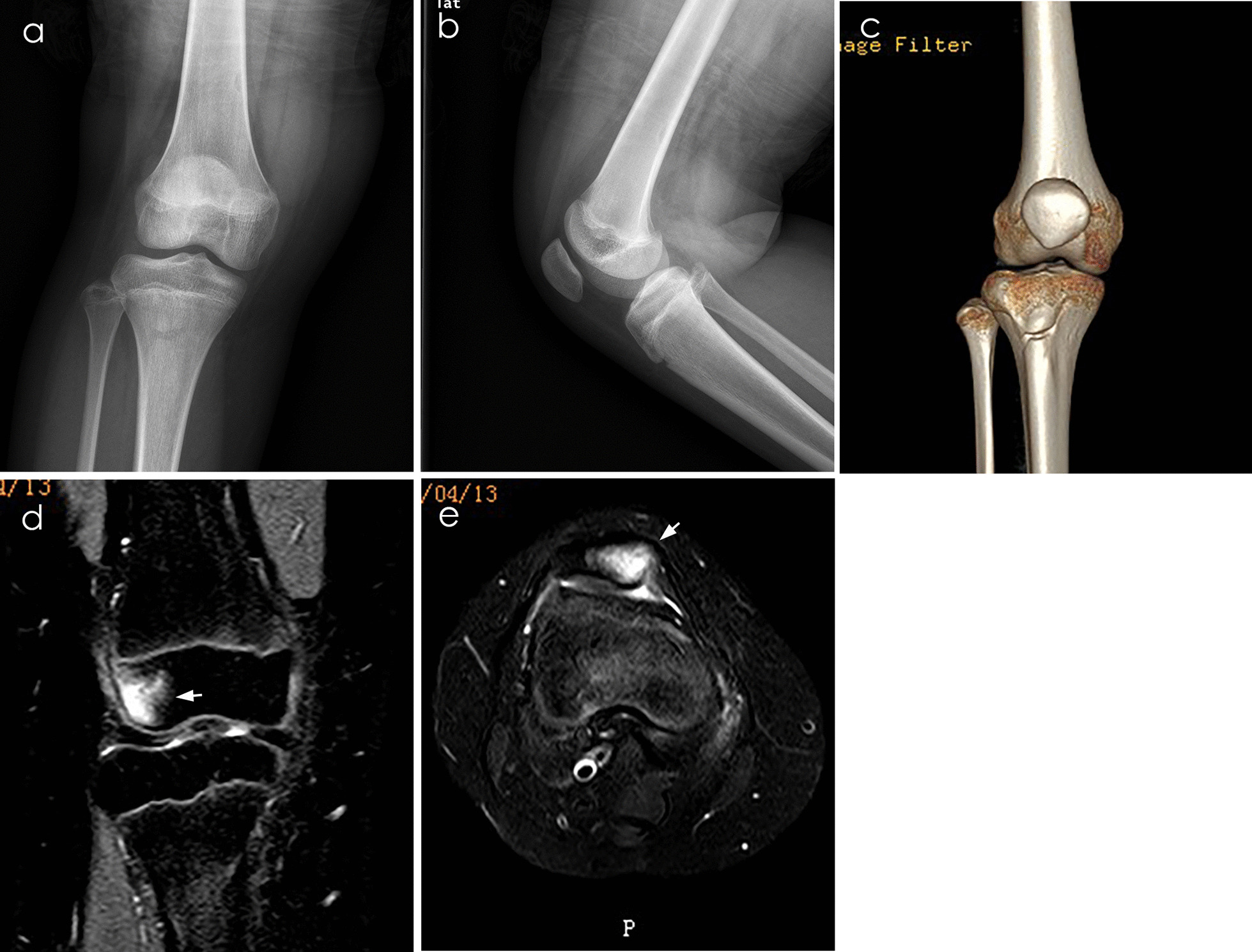
Plain radiograph, CT, and MRI of a 10-year-old girl with right traumatic patellar dislocation. **a, b** Anteroposterior and lateral knee radiograph did not show the patella fracture line. **C** CT after reconstruction did not show the patella fracture line. **d, e** Coronal and cross view of T2-weighted MRI showed bone contusion on the medial side of the patella of the lateral femoral condyle

Our hypothesis was that there would be clinically significant differences in knee swelling-related physical examination and SP measurement in X-ray examination between the neglected OCFs group and the osteochondral contusion only group (Fig. 4c, d, e). In this retrospective study, we compared the potential risk factors in these two groups, which may help to predict the possibility of neglected OCFs. These results could show when further CT/MRI examination is needed and reduce medical costs and use of radiation.

## Materials and method

Following ethical approval from the institutional review board at our hospital, we reviewed the clinical and radiological records of all children with primary ATPD between January 2010 and February 2022, retrospectively.

### Inclusion criteria

Patients were included if (1) they had primary ATPD within four days, (2) they were under 18 years old, and (3) complete clinical and radiological examination were available. This patellar misalignment is diagnosed on the basis of the trauma history and clinical findings. Some patients may remember feeling the patella in a laterally displaced position. Clinical findings include knee hematoma, a lateralized patella and pain at the site of the medial restraints [22].

Neglected fracture in ATPD is defined as meeting three criteria simultaneously if (1) either our hospital or other hospitals had not diagnosed neglected fractures on initial X-ray,(2) at least one of the three authors (MM, LH, and HC) could not confirm the existence of fractures on X-ray, and (3) confirmed the osteochondral fractures through subsequent CT or MRI.

### Exclusion criteria

Patients were excluded if (1) there was recurrent/ habitual patellar dislocation or other patellar malalignment; (2) there was obvious fracture on X-ray; (3) fractures were present that did not come from the patellofemoral joint; or (4) there was neither CT nor MRI examination.

Acute knee injury was evaluated by pediatric orthopedic surgeon (Table 1). Knee radiography was completed in all patients. Medical history, physical examination, and radiographic records were reviewed to confirm primary ATPD. Post-ATPD pain, swelling, and fear can affect the walking and gait, which needs to be evaluated and divided into four levels (Table 1).

**Table 1:** Summary of demographic and clinical data of all subjects

Characteristics	Neglected fracture group	Without fracture group	*p* Value
	(*N* = 79)	(*N* = 34)	
Gender (male/female)	38/41	9/25	0.032
Side (left/right)	45/34	17/17	0.495
Age (months)	159.4 ± 16.4	156.1 ± 25.3	0.399*
Weight (kg)^#^	63 (33–131)	52.5 (31–74)	< 0.001*
Knee injury pattern			0.904
Sprains	50	23	
Falls	24	9	
Direct violence	5	2	
Displacement sensation			0.282
Yes	45	17	
No	17	12	
Uncertain	17	5	
Reduction mode			0.904
Spontaneous	49	25	
Patient-themself	17	7	
Others	13	2	
Walking ability			< 0.001
Normal	0	6	
Mild limp	7	16	
Obvious limp	30	10	
Inability	42	2	
Effusion grade			< 0.001*
1	11	24	
2	46	8	
3	19	2	
4	3	0	
SP (mm)	24.6 ± 4.5	13.2 ± 3.2	< 0.001

Numbers displayed are mean ± standard deviation.

All calculated by the Chi-square test, except the age, weight, and SP measurement, which were calculated by independent samples test*.Values of weight measurements as medians (interquartile range)^#^.

The SP was manually compressed, forcing the joint effusion distally. Knee effusion can be graded in size by compressing the SP. If any fluid was noted, it was scored as knee effusion grade 1, slight lift-off of the patella was scored as grade 2 and a ballotable patella as grade 3, while grade 4 represented a tense effusion with no ability to compress the patella against the femoral sulcus [23]. The data of knee examination were extracted from the medical records.

The greatest dimension of the SP effusion on lateral knee radiograph was measured and documented [21]. This measurement is defined by obtaining the anteroposterior distance from the anterior edge of the pre-femoral fat pad to the posterior edge of the SP fat pad (Fig. 1b) [24, 25]. Further imaging examination is decided on by the surgeon.

ATPD with neglected OCF was defined as Group A, while ATPD without OCF was defined as Group B. Group A was divided into two subgroups according to the maximum fracture diameter. Three-dimensional diameters of the OCF were measured and the longest diameter were recorded. For osteochondral defects with a size greater than 10 mm, they have a better prognosis if the fragment received reduction and fixation [5]. Group A1 was defined as greater than or equal to 10 mm, which were considered severe fractures, and Group A2 was defined as having fractures of less than 10 mm.

The primary endpoint is neglected OCFs. The main aim of study is to confirm risk factors of neglected OCFs in primary ATPD. So, the sample size we enrolled need to present significant difference between Group A and Group B at least.

Data analyses were performed using SPSS software (SPSS for Windows, v. 20.0; SPSS, Inc., Chicago, IL). Data were shown as mean ± standard deviation). An independent samples test was performed to compare the differences between the two groups. We performed the test of normality for the age, weight, and SP measurement through Kolmogorov–Smirnov test. Non-parametric distributions data were performed by Kruskal–Wallis test in our study. A Chi-square test was used to analyze the frequency data on gender, side, knee injury pattern, displacement sensation, reduction mode, walking ability, effusion grade, and fracture site. Multivariable linear regression analysis was applied to detect the effect of other independent variables on the OCF in ATPD and fragment size.

The receiver operating characteristic (ROC) curve for the risk factor for neglected OCFs was plotted to determine the optimum cutoff point, and area under the ROC curve (AUC) was used to determine the discrimination power between Group A and Group B. The same were used to compare Group A1 and Group A2. Statistical significance was defined as values of *p* < 0.05.

## Results

A total of 113 children (113 knees) met the inclusion criteria, of whom 79 were in Group A and 34 were in Group B. Two and one patients were excluded due to accompanied tibial intercondylar spine fracture and tibial tubercle fractures, respectively. Eleven patients were excluded due to lack of CT/MRI examination. Most neglected OCFs (68/79) were not confirmed by all three observers. The demographic and clinical data of all enrolled patients are reported in Table 1. Weight was considered non-parametric distributions, so the Kruskal–Wallis test was performed. We have displayed the values of these measurements as medians (interquartile range) in Table1.

### Risk factors associated with neglected fracture

There was significant difference in gender, weight, walking ability, effusion grade, and SP measurement in Group A and Group B (gender: *p* = 0.032; weight: *p* < 0.001; walking ability: *p* < 0.001; effusion grade: *p* < 0.001; and SP measurement: *p* < 0.001) (Table 1).

When multivariate linear regression analysis was used, after controlling for the compounding factors of gender, weight, walking ability, effusion grade, and SP measurement, the difference between Group A and Group B in weight, walking ability, effusion grade, and SP measurement remained statistically significant (weight: *p* = 0.046; walking ability: *p* < 0.001; effusion grade: *p* = 0.048; SP measurement *p* < 0.001) (Table 2).

**Table 2:** Neglected fracture in acute traumatic patellar dislocation and stepwise model multivariable associations

Parameters	Neglected fracture	
	Beta	*P* Value
Weight	0.004	0.046
Walking ability	− 0.143	< 0.001
Effusion grade	0.091	0.048
Suprapatellar pouch measurement	− 0.057	< 0.001

**Fig. 5:**
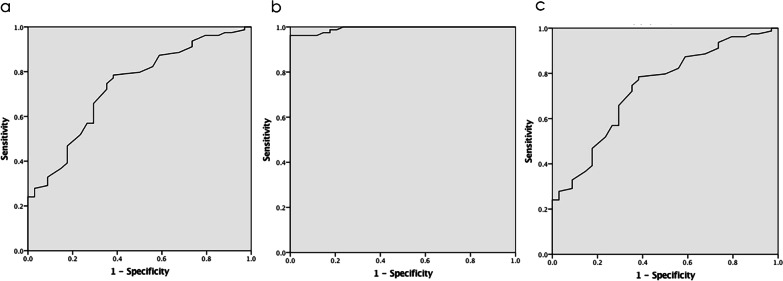
**a** Characteristic curve of body weight as a factor related to neglected fracture. The area under the curve was 0.733 (confidence intervals: 0.635–0.831). **b** Characteristic curve of suprapatellar pouch measurement as a factor related to neglected fracture. The area under the curve was 0.993 (confidence intervals: 0.984–1.000). **c** Characteristic curve of suprapatellar pouch measurement as a factor related to fragment size. The area under the curve was 0.748 (confidence intervals: 0.640–0.856)

The power of neglected fracture variables to differentiate between Group A and Group B. The ROC curves for weight and neglected fracture showed that the cutoff point was 53.5 for weight and 18.45 for SP measurement between the two groups. The AUCs for weight (0.733) and SP measurement (0.993) showed a greater power to differentiate between the two groups (Fig. 5a, b).

### Risk factors associated with fracture fragment size

In two of the 79 patients in Group A, it was difficult to accurately measure the fracture diameter because only the fracture line was seen without obvious displacement. Therefore, the remaining 77 cases were included in the risk factors related to fragment size. All 77 cases received CT examination and the OCF size was measured by CT.

The mean fracture fragment size (longest diameter) in 77 patients was 12.5 ± 5.0 mm; 63 patients had one fracture fragment, and 14 patients had two fracture fragments. Fracture location was also calculated, and 24, 31,11, four, and seven cases were from medial patella, medial inferior margin of patella, inferior margin of patella, femoral condyle, and both patella and femur, respectively.

The results of the Spearman's correlation analysis of the fracture fragment size and potential risk factors are shown in Table 3. Gender, weight, effusion grade, SP measurement, and number of fracture pieces were all significantly related to the fracture fragment size (gender: *p* = 0.031; weight: *p* = 0.006; effusion grade: *p* = 0.013; SP measurement: *p* < 0.001; number of fracture pieces: 0.042) (Table 3).

**Table 3:** Spearman correlation calculated for variation in neglected fracture group (*n* = 77)

Parameters	Fracture length	
	*r*	*p* Value
Gender	**− ** 0.246	0.031
Side	0.119	0.301
Age	**− **0.121	0.296
Weight	0.313	0.006
Knee injury pattern	0.151	0.190
Displacement sensation	0.173	0.133
Reduction mode	0.023	0.840
Walking ability	**− **0.027	0.813
Effusion grade	0.281	0.013
SP measurement	0.411	< 0.001
Number of fracture fragments	0.232	0.042
Fracture site	0.005	0.965

Multivariate linear regression analysis was used, after controlling for the compounding factors of gender, weight, effusion grade, SP measurement, and number of fracture fragments. In the 77 neglected fracture patients, SP measurement remained statistically significant (beta value = 0.457, *p* < 0.001).

The power of variables to differentiate between Group A1 and Group A2. The ROC curves for SP measurement and fragment size showed that the cutoff point was 26.2 for SP measurement between Group A1 and Group A2. The AUCs for SP measurement (0.748) showed a greater power to differentiate between Group A1 and Group A2 (Fig. 5c).

## Discussion

Our results show a significant correlation between weight, walking ability, effusion grade, and SP effusion with neglected OCFs in primary ATPD. This study also demonstrates a significant correlation between SP effusion and fragment size in the Group A.

High incidence of OCFs and low radiographic detection rates have become an issue of concern in ATPD. Seeley and Stanitski reported that 38% and 67% of patients with ATPD had OCFs in adolescents, respectively [10, 26]. However, pediatric orthopedic surgeons have noted the inaccuracy of routine knee radiographs in detecting OCFs (Figs. 1c, 2c). Stanitski et al. reported that arthroscopically documented osteochondral injury was radiographically identified in only 34% of adolescents, only 29% osteochondral loose bodies were radiographically identified, and identification of this osteochondral injury was independent of fragment size [10]. Dainer et al. found osteochondral defects greater than 5 mm that were not evidenced radiographically in 40% of patients [27]. In this study, OCFs were found in 70% of ATPD through further imaging examination which it had not been possible to confirm on knee radiography. For those patients who do have surgical indications, immediate treatment provides the highest gain in quality-adjusted life years and is the preferred cost-effective strategy [28]. Therefore, it is particularly important to decide which patients need further CT and MRI examination.

Few studies have described the OCFs and detailed predisposing factors, particularly in a general pediatric population. Uimonen et al. found that OCF patients had an increased tibial tubercle-posterior cruciate ligament distance and more substantial patella lateralization [29]. Nietosvaara et al. found that fragments were found only after spontaneous relocation of the patella [30]. In this study, four risk factors are associated with neglected OCFs. In this study, body weight greater than 53.5 kg is considered to be associated with significantly increased OCFs. The proportion of obvious limps and inability to walk in Group A was higher than that in Group B (72/79 versus 12/34). Similarly, the proportion of obvious effusion grade in Group A was higher than that in Group B (22/79 versus 2/34). Knee hemarthrosis and medial patellofemoral ligament injury can lead to limited load, higher effusion grade, and SP effusion (Figs. 2f and 3e). Kastelein et al. reported that the combination of history taking and physical examination was of diagnostic value in knee injury [17].

In this study, wider SP measurement is not only a risk factor for neglected OCFs but also predicts fragment size. Concomitant OCFs with disrupted cortical bone are associated with a higher degree of joint inflammation and effusion [31]. Hemarthrosis and exudation within the SP following OCFs can lead to significantly restricted range of motion and walking ability. Kaneko et al. reported that most knees had effusions in the SP with traumatic knee disorders through MRI [32]. Hall reported that the fat pad separation sign which corresponds to the width of the SP was the most accurate indicator of fluid and effusions [24]. Tai et al. reported that qualitative visual SP assessment of lateral knee radiographs is highly sensitive and accurate for the detection of joint effusion compared with MRI [20]. Cecava et al. reported that SP measurement greater than 10 mm in lateral knee radiography should prompt consideration of further examination in acute knee injury [21]. In this study, we found that an SP measurement of more than 18.45 mm should be considered as requiring further radiological examination to confirm neglected OCFs. An SP measurement of more than 26.2 mm indicates a greater possibility of large fracture fragments. The size of OCF fragments is an important factor affecting surgical decision-making in ATPD [5].

Generally, the younger population is more susceptible to OCFs in ATPD because of a combination of ligamentous laxity and an abrupt change at the interface between articular cartilage and subchondral bone [5]. In this study, there was no statistical difference between the mean injury ages between the two groups. Previous studies have found that OCF fragments mainly come from the patella; similarly, this proportion in our study is 85.7% (66/77).

Certain limitations in this study should be considered. Firstly, this is a retrospective study, and it is difficult to avoid selection bias. Secondly, theoretically minimal knee flexion helps in adequately visualizing SP in lateral radiographs [24]. However, patients may not be able to fully achieve the standard position for pain and have limited knee activity. Thirdly, patellar dislocation recurrence and subsequent treatment outcomes were not analyzed. This study focused on the risk factor of OCF diagnosis. Fourthly, based on the results of this study, we have improved the further imaging examination process but did not compare the effectiveness difference before and after the improved diagnostic process. Fifthly, we did not use non radiative ultrasound to evaluate knee visualize and assess knee effusion and bone erosions.

## Conclusion

This may be the first known consecutive series study in the English-language literature examining risk factors of neglected OCFs within primary ATPD in the adolescent population before CT and MRI scan. Weight, walking ability, effusion grade, and SP measurement are risk factors of neglected OCFs in ATPD. The statistical results of weight and effusion grade are very close to the critical value with statistical significance. SP measurement is also positively correlated with OCF size. We should pay attention to the walking ability and SP measurement on X-ray when receiving primary ATPD patients before administering CT and MRI scans.

## Data Availability

The datasets are available from the corresponding author on reasonable request.

## Abbreviations

ATPD: Acute traumatic patellar dislocation
SP: Suprapatellar pouch
OCFs: Osteochondral fractures
CT: Computed tomography
MRI: Magnetic resonance imaging
